# H3K4me3-Mediated Upregulation of LncRNA-HEIPP in Preeclampsia Placenta Affects Invasion of Trophoblast Cells

**DOI:** 10.3389/fgene.2020.559478

**Published:** 2020-12-11

**Authors:** Ningxia Sun, Huaiyan Chen, Yan Ma, Wenjuan Pang, Xiang Wang, Qing Zhang, Lu Gao, Wen Li

**Affiliations:** ^1^Department of Reproductive Medicine, Changzheng Hospital, Second Military Medical University, Shanghai, China; ^2^Department of Physiology, Second Military Medical University, Shanghai, China; ^3^School of Medicine, International Peace Maternity and Child Health Hospital, Shanghai Jiao Tong University, Shanghai, China; ^4^Shanghai Key Laboratory for Assisted Reproduction and Reproductive Genetics, Shanghai, China

**Keywords:** lncRNA, placenta, preeclampsia, hypoxia, H3K4me3

## Abstract

Preeclampsia (PE) is a pregnancy-related disease defined as onset of hypertension and proteinuria after the 20th week of pregnancy, which causes most maternal and perinatal morbidity and mortality. Although placental dysfunction is considered as the main cause of PE, the exact pathogenesis of PE is not yet fully understood. Long non-coding RNAs (lncRNAs) are implicated in a broad range of physiological and pathological processes, including the occurrence of PE. In this study, we investigated the expression and functions of HIF-1α pathway–related lncRNA-HEIPP (high expression in PE placenta) in the pathogenesis of PE. The expression of lncRNA-HEIPP in the placenta from women who underwent PE was screened by lncRNA microarray and then verified using real-time polymerase chain reaction. Then, the methylation profile of the *lncRNA-HEIPP* promoter and the enrichment of H3K4me3 binding were assessed by bisulfite pyrosequencing and chromatin immunoprecipitation (ChIP)–quantitative polymerase chain reaction (qPCR) assay, respectively. It was found that the level of lncRNA-HEIPP in the PE placenta was significantly higher than that in normal placenta and was increased in HTR-8/SVneo human trophoblast cells upon hypoxia treatment. Moreover, we reported that H3K4me3 manifested significantly higher promoter occupancy on *lncRNA-HEIPP* promoter in HTR-8/SVneo cells upon hypoxia treatment and found that the downregulation of lncRNA-HEIPP promoted trophoblast invasion. Our findings suggested that the hypoxia-induced expression of lncRNA-HEIPP mediated by H3K4me3 modification in trophoblast may contribute to the pathogenesis of PE.

## Introduction

Preeclampsia (PE) is a common complication of human pregnancy defined as the occurrence of hypertension and significant proteinuria after the 20th week of gestation, occurring in approximately 3 to 5% of all pregnancies and resulting in about 50,000 maternal deaths annually worldwide (Gormley et al., 2017). Preeclampsia is also the leading cause of fetal morbidity and mortality (Mayrink et al., 2018). It is characterized by maternal arterial pressure >140/90 mmHg and proteinuria >0.3 g/24 h after 20 weeks’ gestation (Armaly et al., 2018). Current studies suggested that the pathogenesis of PE is closely related to immune system and genetic dysfunctions (Hosseini et al., 2018; Yong et al., 2018). However, the dysfunction of placenta plays a central role in the pathogenesis of PE (Aouache et al., 2018; Gutierrez et al., 2019).

Recent studies have shown that abnormal trophoblast differentiation, migration, and apoptosis often cause decreased invasion of trophoblast cells and remodeling of uterine spiral artery, resulting in reduced uteroplacental perfusion (Cotechini et al., 2014; Chen and Khalil, 2017; Brosens et al., 2019). This further leads to hypoxia and inflammatory changes in the placenta, which will eventually lead to PE (Harati-Sadegh et al., 2018; Tong and Giussani, 2019). Recent studies have shown that the important phenotypes of PE, such as hypertension and proteinuria, are closely related with hypoxia inducible factor-1α (HIF-1α) (Wang et al., 2017; Alici Davutoglu et al., 2018). HIF-1α is a transcription factor that controls the expression of hypoxia-induced factors such as vascular endothelial growth factor (VEGF), platelet-derived growth factor, and so on (Kuzmanov et al., 2012; Zhang et al., 2019), which are considered to play important roles during the pathogenesis of PE. Recently, it has been found that long non-coding RNA (lncRNA) could regulate the invasion and apoptosis of trophoblast cell lines (Song et al., 2017; Xu et al., 2018), suggesting their potential roles in PE occurrence. However, whether hypoxia and HIF-1α regulate the expression of specific lncRNAs during PE has not been investigated yet. So, the objective of the current study is to investigate the differentially expressed lncRNAs in human placenta between normal pregnancy and PE and dissect the mechanisms underlying the regulation of lncRNAs by hypoxia as well as their functions in trophoblast cells migration.

## Materials and Methods

### Patient Samples

All patients provided written informed consent, and this study was approved by the Human Research Ethics Committee of the Shanghai Changzheng Hospital at the Second Military Medical University. Thirty PE patients (mean age of 33.20 ± 0.46 years) and 30 control patients (mean age of 32.60 ± 0.47 years) who received a cesarean section were included in this study. Preeclampsia was clinically diagnosed before cesarean. The main criteria for PE diagnosis were as follows: systolic blood pressure ≥140 mm Hg or diastolic blood pressure ≥90 mm Hg on two occasions at least 4 h apar, and proteinuria >0.3 g/24 h. The control pregnancy was defined as no medical complications and proteinuria, and maternal blood pressure <140/90 mm Hg. Clinical characteristics and detailed information of all patients enrolled in this study are summarized in Table 1. The original demographic data of enrolled patients can be found in Supplementary Table 1. The placenta specimen was resected from the middle of villous lobule, avoiding any visible blood clot or calcification. To minimize blood contamination, each piece of tissue was intensively washed with ice-cold phosphate-buffered saline and then immediately snap-frozen in liquid nitrogen after resection. Of the 30 pairs of placenta samples, four pairs were randomly selected for lncRNA and mRNA microarray analysis, and the other samples were used for the following verification.

**TABLE 1:** Clinical characteristics of the pregnant women enrolled in this study^a^.

	Normal (*n* = 30)	Preeclampsia (*n* = 30)	*P-*value
Maternal age (y)	32.60 ± 0.47	33.20 ± 0.46	0.368
Gestational age (wk)	38.35 ± 0.34	34.44 ± 0.15^b^	<0.001
BP (mm Hg)			
Systolic	108.13 ± 0.81	163.87 ± 1.30^b^	<0.001
Diastolic	70.17 ± 0.85	104.43 ± 1.23^b^	<0.001
Proteinuria (g/24 h)	N/A	2.62 ± 0.20	N/A
Fetal birth weight (g)	3,278.00 ± 58.01	2,577.33 ± 97.73^b^	<0.001

*BP, blood pressure. ^*a*^Data are shown as mean ± SEM. ^*b*^Compared with normal pregnancy: *P* < 0.001.*

### RNA Extraction

Total RNA was extracted from snap-frozen placenta tissues obtained from PE placenta patients and control patients using TRIzol Reagent (Invitrogen, Carlsbad, CA, United States) according to the manufacturer’s instruction. The RNA integrity and concentration were evaluated using the NanoDrop ND-1000 spectrophotometer and 2100 RNA Nano 6000 Assay Kit (Agilent Technologies, Santa Clara, CA, United States).

### RNA Microarray and Computational Analysis

RNA with RNA integrity number greater than 6.5 purified from total RNA following the removal of rRNA was amplified and transcribed into fluorescent cDNA along the entire length of the transcripts without 3′ bias utilizing random priming method, and cDNA was labeled and hybridized to the Human LncRNA Array V2.0 (8 × 60, Arraystar). In addition, 30,215 coding transcripts were detected using the microarray as well. The microarray was performed by KangCheng Bio-tech (Shanghai, China). The arrays were scanned using the Agilent Scanner G2505B (Agilent Technologies), and the acquired array images were analyzed using Agilent Feature Extraction software (version 11.5.1; Agilent Technologies). Quantile normalization and subsequent data processing were performed using GeneSpring GX v11.5.1 software package (Agilent Technologies).

### Gene Ontology Pathway Analysis

To investigate the roles and associated pathways of differentially expressed lncRNAs and mRNAs, Gene Ontology (GO) pathway analysis was performed to annotate the transcripts with terms under the biological process, cellular component, and molecular function ontologies. GO annotations of microarray genes were downloaded from NCBI^1^, UniProt^2^, and the GO^3^. A Fisher exact test was performed in order to locate the significant enrichment pathway. The resulting *P*-values were adjusted using the Benjamini–Hochberg false discovery rate (BH FDR) algorithm. Pathway categories with a FDR < 0.05 were reported.

### Quantitative Reverse Transcription Real-Time Polymerase Chain Reaction

Total RNA (2 μg) was reverse transcribed with random hexamers using the Reverse Transcription System Kit (Promega Corporation, Madison, WI). LncRNA expression in placenta tissues was quantified using a standard quantitative reverse transcription real-time PCR (qRT-PCR) on the StepOne Plus system (Applied Biosystems, Foster City, CA, United States), in a total reaction volume of 20 μL, including 10 μL SYBR Premix Ex Taq (2x), 1 μL of PCR Forward Primer (10 uM), 1 μL of PCR Reverse Primer (10 uM), 2 μL of cDNA, and 6 μL of double-distilled water. Primer sets are listed in Supplementary Table 2. The qRT-PCR was performed with an initial denaturation step of 10 min at 95°C; 95°C (15 s), 60°C (30 s), 72°C (30 s) for a total 40 cycles; and a final extension step at 72°C for 5 min. All experiments were performed in triplicate. All samples were normalized to GAPDH. The geometric mean in each triplicate was used to calculate the relative lncRNAs concentrations (ΔCt = Ct of lncRNAs − Ct of GAPDH). The fold change of expression was calculated using the 2^–Δ^ ^Δ^ ^Ct^ method.

### Cell Line and Hypoxia Treatment

The immortalized EVT cell line HTR-8/SVneo was used. The cells were maintained in RPMI-1640 supplemented with 5% fetal calf serum, 1% L-glutamine 200 mM, and 1% penicillin–streptomycin (all from Invitrogen) under an atmosphere of 5% CO_2_ at 37°C. To establish the hypoxia model, the cells were exposed to 94%N_2_/1% O_2_/5% CO_2_ for 2, 24, 48, and 72 h. The control group was cultured under normal air condition for the corresponding period.

### HIF-1α Overexpression in HTR-8/SVneo Cells

The coding sequence of human HIF-1α was amplified by PCR from human genomic DNA libraries (GENECHEM Company, Shanghai, China) and was subcloned into the pEZ-MO2 plasmid via homologous recombination according to manufacturer’s protocol (Hieff Clone^®^ Plus One Step Cloning Kit, YEASEN, Shanghai, China) to construct pEZ-MO2–HIF-1α overexpression. The sequence of the construct was confirmed by DNA sequencing at Sangon Biotech Company (Shanghai, China). The HTR-8/SVneo cells at 70% confluence were transiently transfected with pEZ-MO2-HIF-1α plasmid for 48 h using Lipofectamine 3000 (Invitrogen) according to manufacturer’s instructions. The empty pEZ-MO2 plasmid was also transfected as negative control (NC).

### Small Interfering RNA Transfection

lncRNA-HEIPP in HTR-8/SVneo cells were knocked down by transfection of preannealed stealth small interfering RNA (siRNA) duplexes designed online by Thermo Fisher’s BLOCK-iT^TM^ RNAi Designer^4^, using Lipofectamine RNAiMAX transfection reagent (Invitrogen). *Silencer*^®^ Negative Control #1 siRNA (si-NC, AM4611, Ambion) was transfected as an NC. The efficiency of knockdown was determined by RT-PCR.

### Cell Counting Kit-8 Cell Proliferation Assay

Cell viability was assessed using the Cell Counting Kit-8 (Dojindo Laboratories, Kumamoto, Japan) according to the manufacturer’s protocol. Cell proliferation curves were plotted using the absorbance at each time point (0, 12, 24, and 48 h). All experiments were performed in triplicate.

### Matrigel Invasion Assay

The invasion of HTR-8/SVneo cells across Matrigel was objectively evaluated in an invasion chamber according to the manufacturer’s protocol (Millipore). The cells transfected with si-NC or si-HEIPP (10 and 30 μL) were plated in the upper chambers of 24-well plates at a density of 1.0 × 10^4^ cells/well in RPMI-1640 medium containing 5% fetal bovine serum; the membrane pore of the Transwell chamber was 8 μm in diameter. The membranes were coated with Matrigel (BD, United States) to form matrix barriers at the concentration of 200 μg/mL. A 700-μL aliquot of RPMI-1640 medium containing 10% FBS was immediately placed in the lower well of the chamber as a chemoattractant. After incubation for 48 h at 37°C, cells were completely removed from the upper surface of the filter using a sterile cotton swab, and cells that had migrated to the lower surface were fixed and stained with 0.1% crystal violet. Finally, the cell migration ability was determined by counting the number of stained cells on the membranes in 10 randomly selected, non-overlapping fields at 20× magnification under microscope. The migration index was calculated as the ratio of the percentage of cell migration in the various treatments to that of the vehicle. Each experiment was performed in triplicate and repeated three times.

### Bisulfite Pyrosequencing

Bisulfite pyrosequencing was used to quantity DNA methylation at two sites in the DMR of the lncRNA-HEIPP gene. Two micrograms of DNA was subjected to bisulfate conversion using the EpiTect Bisulfite Kit (59104) (Qiagen Ltd., GmbH, Hilden, Germany). Pyrosequencing primers were designed using PyroMark Assay Design 2.0 (Qiagen Ltd., GmbH, Hilden, Germany) and listed in Supplementary Table 2. One hundred nanograms of bisulfite-converted DNA was PCR amplified in a 50-μL reaction volume containing 5 × PCR buffer (KAPA Ltd., United States), 10 mM of each dNTP (KAPA Ltd., United States), 50 mM forward and reverse primers (BGI Inc., Shanghai, China), and 1 U DNA polymerase (HotStart Taq, KAPA Ltd). The PCR cycling parameters were 95°C for 3 min; 40 cycles of 95°C for 30 s, a variable annealing temperature (Ta) for 30 s, and 72°C for 1 min; and extension at 72°C for 7 min. A 25-μL aliquot of each PCR product was subjected to pyrosequencing using the PyroMark Q96 ID Pyrosequencer (Qiagen Ltd.) following the manufacturer’s recommended protocols. The degree of methylation at each CpG site was determined using Pyro Q-CpG Quantification software. All samples were analyzed in triplicate.

### ChIP Assay

The HTR-8 trophoblast cell line was used for ChIP-based analysis for enrichment of H3K4me3 on *lncRNA-HEIPP* promoter. ChIP assays were conducted according to the manufacturer’s instruction (Millipore, 17-10086). The chromatin extracted from cells was sheared, subjected to immunoprecipitation with H3K4me3 (Abcam) or immunoglobulin G (IgG) (Millipore) antibodies, reverse cross linked, and subjected to quantitative ChIP- PCR (qChIP). The qChIP was performed on sheared DNA with following primers: SP1: F: 5′ ATTTTGCTTCC TATCCCT 3′, R:5′ ACCGACAATCTCCTCAGT 3′, SP2: F: 5′ GA CTGAGGAGATTGTCGGT 3, R: 5′ CTTATGATGGTCTGGGT GA 3′, SP3: F: 5′ TGGAGGAGGGAGATGACA 3′, R: 5′ TGGA GGAGGGAGATGACA 3′, SP4: F: 5′ CATGCCCTCTCAGC CTAAC 3′, R: 5′ AGTCCCCCCAAGAAAAACA 3′. The four pairs of primers amplified −324 to −1,288 bp region upstream from the transcriptional starting site of lncRNA-HEIPP. The results were normalized to input and expressed as a percentage of the fold difference. IgG was used as an NC. Data were obtained from at least three independent experiments.

### Statistical Analysis

All data are expressed as mean ± SD (standard deviation), and all statistical analyses were performed using the SPSS statistical software package (SPSS, Inc., Chicago, IL, United States). All the data were tested for homogeneity of variance by Bartlett test before analyzing the significance. Comparisons were made using Student *t*-test and analysis of variance. *P* < 0.05 was considered statistically significant.

## Results

### Differential Expression of lncRNAs in Placenta Between PE and Normal Patients

The demographic data of PE women and normal pregnant women are summarized in Table 1. There was no significant difference in maternal age between normal pregnancies and pregnancies with PE (*P* > 0.05). The systolic and diastolic blood pressure was significantly higher in pregnant women with PE (*P* < 0.001). The average proteinuria is 2.62 ± 0.20 g/24 h, which means that the cases enrolled this study were severe PE patients (proteinuria >2.0 g/24 h). Women from the PE group delivered earlier than normal pregnant women mostly due to the medical termination of the pregnancy, which is the major cause of the lower infant birth weight in the PE group, as there was no intrauterine growth restriction patient in the PE group enrolled in this study.

**FIGURE 1:**
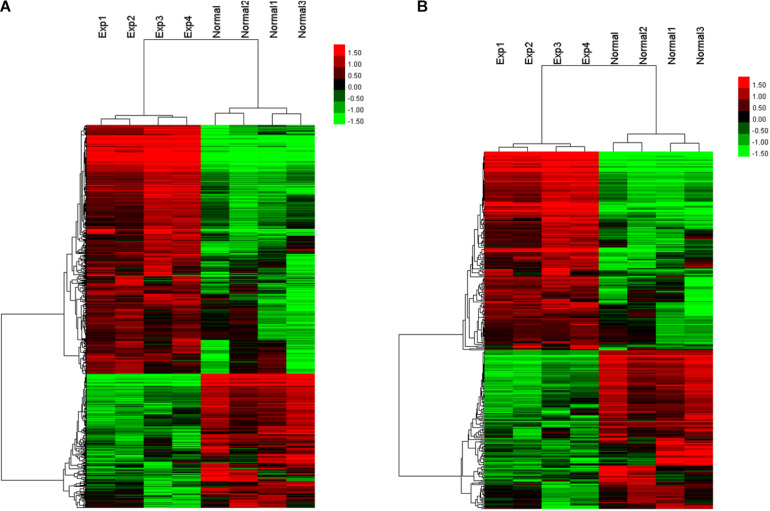
Heatmap and hierarchical clustering of lncRNA expression **(A)** and mRNA expression **(B)** between the preeclampsia placenta (Exp) and normal samples (Normal).

As the transcriptome of the placenta is dynamically changed during advancing gestation (Deyssenroth et al., 2017), to ensure the identical or similar baselines between the two disease groups, we selected four pairs of PE patients and controls with matched gestational age within 2 weeks (Supplementary Table 1). We aligned lncRNA array data into RefSeq_NR, UCSC, and Ensembl database and compared the lncRNA expression levels between four human PE placenta (P) and four normal samples (N). The expression profiles of lncRNAs were shown by calculating the log-fold change (P/N). Among these lncRNAs, 405 were consistently upregulated, and 344 lncRNAs were consistently downregulated (Figure 1A). The clustering analysis also showed the differential expression patterns of mRNAs between PE placentas and normal samples (Figure 1B). The complete dataset regarding the significantly upregulated and downregulated lncRNA or mRNA was deposit on the public repository^5^.

### GO Pathway Analysis

**FIGURE 2:**
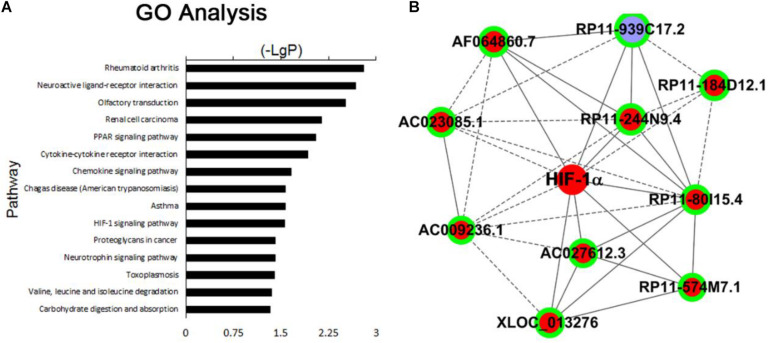
**(A)** The top 15 signaling pathways that were most significantly enriched in PE placenta compared with normal placenta. **(B)** Top 10 lncRNAs related with HIF-1α pathway: AF064860.7, AC027612.3, RP11-244N9.4, RP11-574M7.1, RP11-80I15.4, RP11-939C17.2, XLOC_013276, AC023085.1, AC009236.1, and RP11-184D12.1.

GO pathway analysis was performed to determine the lncRNA and mRNA enrichment in biological processes, cellular components, and molecular functions. HIF-1α pathway is known to play an important role in the pathogenesis of PE and identified as one of the most significant enrichment signaling pathways under our experimental settings (Figure 2A and Supplementary Table 3). We found that a number of lncRNAs and molecules are closely related with HIF-1α pathway (Figure 2B), and the top 10 lncRNAs (AF064860.7, AC027612.3, RP11-244N9.4, RP11-574M7.1, RP11-80I15.4, RP11-939C17.2, XLOC_013276, AC023085.1, AC009236.1, and RP11-184D12.1) showing mostly dramatic different expression between PE and normal placentas were selected for further validation (Supplementary Table 4).

### Validation of Differentially Expressed lncRNAs

**FIGURE 3:**
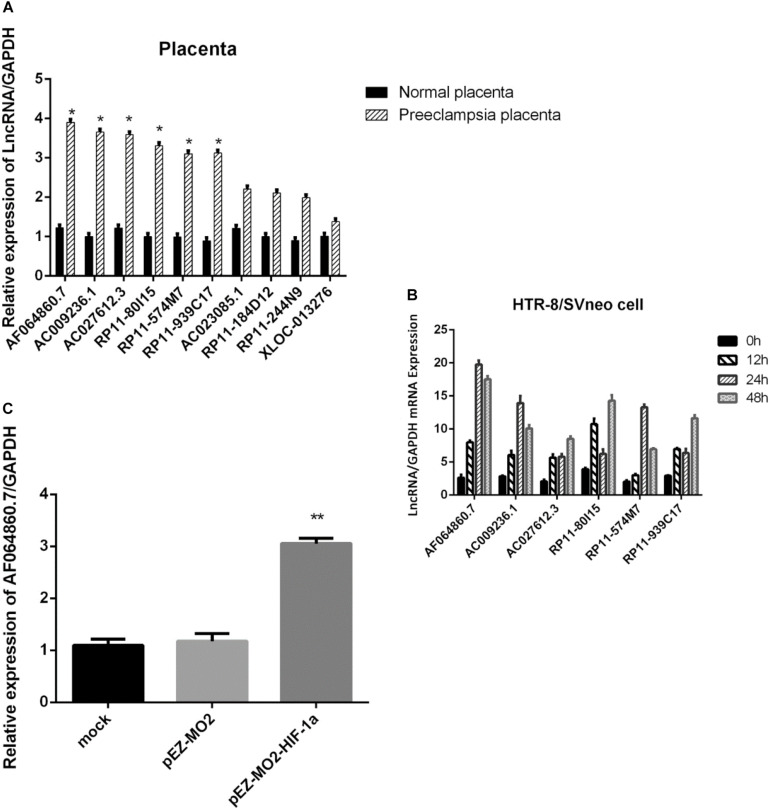
**(A)** Verification of 10 lncRNAs related with HIF-1a pathway in 30 preeclampsia placenta tissues and 30 normal placenta tissues using qPCR. **(B)** Time-dependent changes of the six candidate lncRNA expression upon hypoxia treatment in HTR-8/SVneo cells. **(C)** The overexpression of HIF-1α increased the expression of lncRNA-AF064860.7 (lncRNA-HEIPP) in HTR-8/SVneo cells. *p < 0.05, **p < 0.01.

To further validate the preliminary data from microarray, we performed qRT-PCR to determine the expression of the ten candidate lncRNAs related with HIF-1α pathway in 30 PE placenta tissues and 30 normal placenta tissues. We found that 6 of the 10 lncRNAs manifested significant upregulation in PE samples compared with normal samples (Figure 3A), which are consistent with the array results. Among the six lncRNAs, lncRNA-AF064860.7 showed the most significant differences between PE samples and normal samples, so that we named it lncRNA-HEIPP (high expression in PE placenta).

In the HTR-8/SVneo human trophoblast cell lines, we found that hypoxia could induce the expression of lncRNA-HEIPP, which reached the highest level at 24 h upon hypoxia treatment (Figure 3B). Although the other five candidate lncRNAs also showed time-dependent changes of expression upon hypoxia treatment, the increasing trend and fold change were less significant than that in lncRNA-HEIPP. Moreover, the overexpression of HIF-1α increased the expression of lncRNA-HEIPP, further suggesting the interaction between HIF-1α pathway and lncRNA-HEIPP (Figure 3C).

### Hypoxia Did Not Change the Methylation Profile of lncRNA-HEIPP Promoter in Placental Trophoblast Cells

**FIGURE 4:**
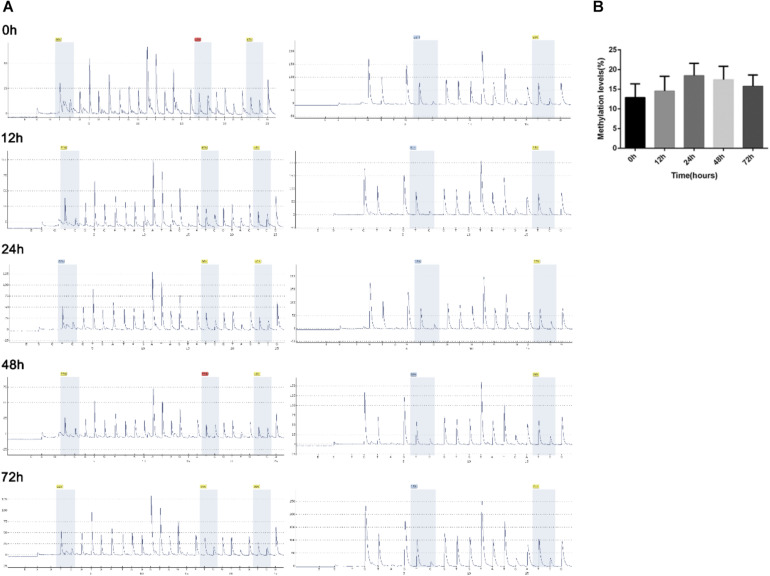
Methylation levels of *lncRNA-HEIPP* promoter in HTR-8/SVneo human trophoblast cells that underwent hypoxia. **(A)** The degree of methylation at each CpG site of *lncRNA-HEIPP* promoter between normoxia (left panel) and hypoxia (right panel) treatment at different time point. **(B)** The statistical graph showing the changes of methylation level of *lncRNA-HEIPP* promoter in HTR-8/SVneo human trophoblast cells that underwent hypoxia at different time point. *n* = 3, and all samples were analyzed in triplicate.

As the epigenetic regulatory elements such as DNA methylation and H3K4me3 occupancy were critical for the gene expression regulation in placental development and trophoblast invasion (Apicella et al., 2019; Kwak et al., 2019), we analyzed the epigenetic regulatory elements of lncRNA-HEIPP in the placenta in PE versus controls. Specifically, compared to the normal oxygen condition, the DNA methylation levels of lncRNA-HEIPP promoter in HTR-8/SVneo human trophoblast cells increased along with the extension of hypoxia time, which reached the highest level at 24 h upon hypoxia treatment (Figure 4A). However, the increase of methylation level was not significant at each time point (Figure 4B).

### Binding of H3K4me3 in *lncRNA-HEIPP* Promoter Was Increased by Hypoxia Treatment

**FIGURE 5:**
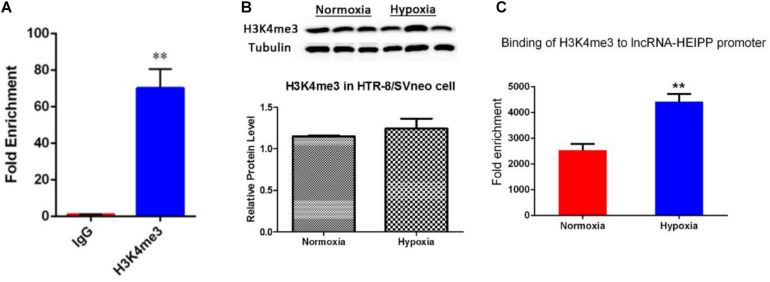
**(A)** ChIP analysis detected the enrichment of H3K4me3 binding on *lncRNA-HEIPP* promoter. **(B)** The protein expression of H3K4me3 in HTR-8/SVneo cells that underwent hypoxia and normoxia. **(C)** The changes of H3K4me3 occupancy on *lncRNA-HEIPP* promoter in HTR-8/SVneo cells that underwent hypoxia compared to those that underwent normoxia, **p < 0.01, *n* = 3.

Enrichment of histone trimethylation at lysine 4 (H3K4me3) at *lncRNA-HEIPP* DMRs/promoters in HTR-8/SVneo human trophoblast cells that underwent hypoxia or normoxia was assessed using ChIP-qPCR. It was shown that the binding of H3K4me3 on *lncRNA-HEIPP* promoter was specifically enriched compared with the binding of IgG (Figure 5A). As shown in Figure 5B, the protein expression of H3K4me3 was not affected by hypoxia treatment. However, H3K4me3 manifested significantly higher promoter occupancy on *lncRNA-HEIPP* promoter in HTR-8/SVneo cells that underwent hypoxia for 24 h compared to those that underwent normoxia (Figure 5C), suggesting the histone modification, instead of the DNA methylation might be the epigenetic pathway that causes the increased expression of lncRNA-HEIPP in the placenta during PE undergone hypoxia.

### Downregulation of lncRNA-HEIPP Promoted Trophoblast Invasion

**FIGURE 6:**
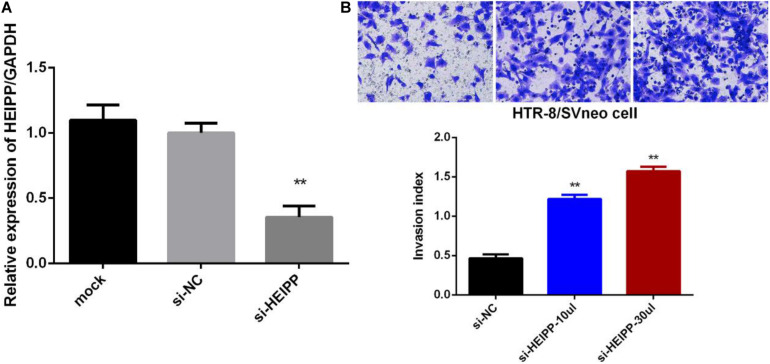
**(A)** Transfection of si-HEIPP significantly knocked down the expression of lncRNA-HEIPP. **(B)** Downregulation of lncRNA-HEIPP promoted HTR8/SVneo trophoblast cells invasion, **p < 0.01.

Next, we assessed the biological functions of lncRNA-HEIPP in trophoblast cells. We knocked down the expression of lncRNA-HEIPP in HTR-8/SVneo human trophoblast cell line using HEIPP siRNA (si-HEIPP) and found that the proliferation of HTR8/SVneo cells was not affected (data not shown). However, the invasion of HTR8/SVneo cells was significantly promoted, compared to the cells treated with NC siRNA (Figure 6).

## Discussion

Preeclampsia is a unique disease during pregnancy. It is a placental syndrome characterized by high blood pressure, edema, and proteinuria after 20 weeks of pregnancy (Gormley et al., 2017; Mayrink et al., 2018). Preeclampsia is the leading cause of perinatal and maternal death. At present, the PE incidence rate is 5–8% throughout the world and is 9.4% in China (Boriboonhirunsarn et al., 2017). Current studies strongly suggested that placenta play central roles in the pathogenesis of PE (Kawasaki et al., 2019). The proliferation and invasion of placental trophoblast cells are the key to successful pregnancy and embryonic development (Knofler and Pollheimer, 2013). The earlier study found that abnormal trophoblast differentiation, migration, and apoptosis often caused the decrease in the invasion of trophoblast cells and the failure of uterine spiral artery recasting, resulting in reduced uteroplacental perfusion and subsequently leading to PE (Kadyrov et al., 2006). However, the factors that affect placental trophoblast cells and the pathogenesis of PE have not been fully elucidated.

In recent years, epigenetic regulation has been revealed to play important roles in the embryonic development and the pathogenesis in many diseases. LncRNAs are a class of transcripts whose lengths exceed 200 nt. Initially, lncRNAs were thought to be transcribed noise. However, an increasing number of studies have reported that these lncRNAs have a series of important functions and participate in the development of many diseases, such as cancer (Liu et al., 2016), cardiovascular illnesses (Leung et al., 2013; Wang et al., 2016), and neurological illnesses (Jiang et al., 2016). Recently, some lncRNAs have been shown to be associated with PE (He et al., 2013; Chen et al., 2015; Zhang et al., 2015). However, their related pathways and regulation were not fully revealed yet. In our current study, we performed lncRNA microarray and found that 1,051 lncRNAs were upregulated, and 344 lncRNAs were downregulated in PE placenta compared with normal placenta, which is comparable to the microarray results from previous study that 738 differently expressed lncRNAs in the placentas of PE in comparison with normal pregnancies (He et al., 2013), but is much less than another study in which 15,646 upregulated and 13,178 downregulated lncRNAs were identified in the placenta tissues of early-onset PE patients (Long et al., 2016). However, in the latter study, the tissues were collected from early-onset PE patients, and the controls were preterm birth, and the biased factor, i.e., preterm labor, may partially explain the huge differences in the numbers of differentially expressed lncRNAs between studies.

The decreased trophoblast proliferation, migration, invasion, and stimulated apoptosis constitute the pivotal reasons leading to PE (Fu et al., 2013). Previous studies revealed different lncRNAs that affect cellular functions of trophoblast cells (Cao et al., 2017; Song et al., 2017; Jiao et al., 2018; Li et al., 2018). However, the hypoxia and HIF-1α–induced pathways play central roles in the pathogenesis of PE (Korkes et al., 2017; Harati-Sadegh et al., 2018; Tong and Giussani, 2019), whereas no HIF-1α–related lncRNAs have yet been revealed in the previous studies. In the current study, we first showed that lncRNAs related with HIF-1α pathway were significantly increased in PE placenta compared to normal placenta, among which lncRNA-HEIPP manifested most significant and time-dependent upregulation upon hypoxia treatment. In tumors, it was found that microRNAs and lncRNAs are important in enabling the key hypoxia-regulated processes (Ferdin et al., 2013; Choudhry et al., 2016). In placenta, some microRNAs, such as microRNA-218 and microRNA-18b, were shown to be induced by hypoxia and HIF-1a in PE patients (Fang et al., 2017; Wang et al., 2017). To our knowledge, lncRNA-HEIPP is the first lncRNA that was found to be upregulated by HIF-1a in placental tissues of PE patients. Moreover, Wang et al. (2018) also found that some lncRNAs, such as NONHSAT116812 and NONHSAT145880, were also confirmed in plasma specimens, making them to be potentially utilized as indicators for PE. Whether lncRNA-HEIPP can also be detected in plasma and used as novel indicators for PE warranted further confirmation.

In recent years, epigenetic regulation of placental gene expression has been considered to play an important role in human trophoblast differentiation (Gamage et al., 2018) and contribute to the onset of PE (Chelbi and Vaiman, 2008; Leavey et al., 2018). The studies showed higher expression levels of death domain-associated protein 6 (DAXX), MMP9, and BCL2 in placenta from PE tissue, and this increased expression was well correlated to promoter demethylation (Wang et al., 2010; Novakovic et al., 2017; Mohammadpour-Gharehbagh et al., 2019). However, a significant number of the observed methylation changes were not associated with corresponding changes in gene expression, and vice versa (Gamage et al., 2018; Leavey et al., 2018), indicating that alternate methods of epigenetic regulation will need to be explored in PE. Hypoxia was shown to alter the epigenetic profile in cultured human placental trophoblasts (Yuen et al., 2013). In this study, the DNA methylation levels of lncRNA-HEIPP promoter manifested increase upon hypoxia treatment, but the increases showed no significance at each time point after hypoxia. However, it was shown that the H3K4me3 occupancy of *lncRNA-HEIPP* promoter was significantly enriched in the PE placenta compared to the normal placenta. H3K4me3 is a histone modification marker that usually activates the gene transcription upon its binding to the promoter regions (Sati et al., 2012; Zhang et al., 2018; Fanucchi et al., 2019). These results suggested that the histone modification, instead of the DNA methylation, might be the epigenetic pathway that causes the increased expression of lncRNA-HEIPP in the placenta during PE.

Furthermore, we demonstrated that lncRNA-HEIPP knockdown promoted the invasion of trophoblasts. LncRNAs seem to contribute to PE via different pathways. LncRNA PRNCR1 promoted the progression of eclampsia by regulating the MAPK signal pathway (Jiao et al., 2018), while LncRNA CCAT1 promotes the progression of PE by regulating CDK4 (Li et al., 2018). As hypoxia usually induced the activation of JAK/STAT3 signaling pathway to promote trophoblast cell viability and angiogenesis in PE (Xu et al., 2017), whether the cellular functions of lncRNA-HEIPP were also mediated by JAK/STAT3 signaling pathway needs to be further investigated.

## Conclusion

Our findings suggest that HIF-1a–related lncRNA-HEIPP is upregulated in the placenta from women who underwent PE mediated by the enriched binding of H3K4me3 in lncRNA-HEIPP promoter. LncRNA-HEIPP hampers the invasion of trophoblast cells and contributes to the pathogenesis of PE. Our results demonstrated that HIF-1a pathway–related lncRNA-HEIPP expression profile is a potentially new biomarker, and the manipulation of lncRNA-HEIPP may provide theoretical basis for prevention and treatment of PE.

## Data Availability Statement

The microarray data has been uploaded to the GEO dataset and the accession number is GSE160888.

## Ethics Statement

The studies involving human participants were reviewed and approved by Human Research Ethics Committee of the Shanghai Changzheng Hospital at the Second Military Medical University. The patients/participants provided their written informed consent to participate in this study.

## Author Contributions

NS, HC, and YM: validation, formal analysis, and investigation. YM: writing – original draft. WP: methodology and investigation. XW and QZ: validation. LG: conceptualization, writing – review and editing, project administration, and funding acquisition. WL: conceptualization, supervision, and funding acquisition. All authors contributed to the article and approved the submitted version.

## Conflict of Interest

The authors declare that the research was conducted in the absence of any commercial or financial relationships that could be construed as a potential conflict of interest.

## Footnotes

1 http://www.ncbi.nlm.nih.gov/

2 http://www.uniprot.org/

3 http://www.geneontology.org/

4 http://rnaidesigner.thermofisher.com/rnaiexpress/rnaiDesign.jsp

5 http://doi.org/10.5281/zenodo.4039476
