# Supplementary material for: H3K4me3-Mediated Upregulation of LncRNA-HEIPP in Preeclampsia Placenta Affects Invasion of Trophoblast Cells
Source: Front Genet. 2020 Dec 11;11:559478. doi: 10.3389/fgene.2020.559478 (PMC7793904; doi:10.3389/fgene.2020.559478)
Supplement: Supplementary file 1 [file Data_Sheet_1.zip › Table S1.DOCX]

**Supplemental Tables**

Table S1**.** Primer sequences used for the validation of the ten candidate lncRNAs and bisulphite pyrosequencing.

| **Gene** | **Primer Sequence PCR product size** |
| --- | --- |
| **AF064860.7** | **Forward:** ACGGGCTGCAAGGTTTTACT 126bp |
|  | **Reverse:** GCAGGACTGCCCCATATGTA |
| **AC027612.3** | **Forward:** CAGTGCCATTTGGAGCATCA 207bp |
|  | **Reverse:** ACAACCATATCGAGCACAGAGT |
| **RP11-244N9.4** | **Forward:** GGCAAGACCCAAATGGACAG 147bp |
|  | **Reverse:** GGGGCTGGAGAACTTGAGTC |
| **RP11-574M7.1** | **Forward:** AGGCTGCACCGTACCAAAAA 109bp |
|  | **Reverse:** CGAGCCTTGGGATTTCATGC |
| **RP11-80I15.4** | **Forward:** CCTCCTGGGTTCATGCCATT 140bp |
|  | **Reverse:** TGCTCTGTCTTTCAGGCTGG |
| **XLOC_013276** | **Forward:** GGAGAATGAGGAGAAGGCGT 257bp |
|  | **Reverse:** CCCCTCCTTTTCTTTGTTCCTA |
| **AC023085.1** | **Forward:** ATGTAACCTCCTGGCCCTCA 223bp |
|  | **Reverse:** GGTGTGTCCTGGGTGTTTGA |
| **AC009236.1** | **Forward:** GGCGCACAGACTCATTAACC 175bp |
|  | **Reverse:** TAACCGGCATTTCCAGGTCC |
| **RP11-184D12.1** | **Forward:** CCTGAAAAGGGGGTCAACCA 83bp |
|  | **Reverse:** CTTTGGCTCAGGTTATGAACTC |
| **RP11-939C17.2** | **Forward:** GCAGGCTTGATCTGGGACTT 152bp |
|  | **Reverse:** CGACCACCCCTTCATCTTCC |
| **GAPDH** | **Forward:** TGAATGGGCAGCCGTTAGGA 171bp |
|  | **Reverse:** GGCGCCCAATACGACCAAAT |
|  |  |
| **Pyro-primer1** | **Forward:** TGAGAGGTGAAAAGGTTAGTTAGTG |
|  | **Reverse:** AACCAACCACCATCTCCA |
|  | **Sequencing:** GGTAGGTTATTTAGATTATTATAAG |
| **Pyro-primer2** | **Forward:** GGTGGAGGAGGGAGATGATA |
|  | **Reverse:** ACCAACCACCATCTCCAAATAA |
|  | **Sequencing:** ATGGTTTTTGGTTAGTTGTA |
| **Pyro-primer3** | **Forward:** TTTTAATTTTAGTAGAGATGGGGTGTG |
|  | **Reverse:** TCCAACAACTATAAACTTTCCCCATTC |
|  | **Sequencing:** TGGGGTGTGTTTTTG |
